# Circle the Cardiac Remodeling With circRNAs

**DOI:** 10.3389/fcvm.2021.702586

**Published:** 2021-06-25

**Authors:** Tiqun Yang, Tianxin Long, Tailai Du, Yili Chen, Yugang Dong, Zhan-Peng Huang

**Affiliations:** ^1^Department of Cardiology, Center for Translational Medicine, Institute of Precision Medicine, The First Affiliated Hospital, Sun Yat-sen University, Guangzhou, China; ^2^National Health Commission (NHC) Key Laboratory of Assisted Circulation, Sun Yat-sen University, Guangzhou, China; ^3^National-Guangdong Joint Engineering Laboratory for Diagnosis and Treatment of Vascular Diseases, Guangzhou, China

**Keywords:** cardiac remodeling, non-coding RNA, circular RNA, heart disease, gene regulation

## Abstract

Cardiac remodeling occurs after the heart is exposed to stress, which is manifested by pathological processes such as cardiomyocyte hypertrophy and apoptosis, dendritic cells activation and cytokine secretion, proliferation and activation of fibroblasts, and finally leads to heart failure. Circular RNAs (circRNAs) are recently recognized as a specific type of non-coding RNAs that are expressed in different species, in different stages of development, and in different pathological conditions. Growing evidences have implicated that circRNAs play important regulatory roles in the pathogenesis of a variety of cardiovascular diseases. In this review, we summarize the biological origin, characteristics, functional classification of circRNAs and their regulatory functions in cardiomyocytes, endothelial cells, fibroblasts, immune cells, and exosomes in the pathogenesis of cardiac remodeling.

## Introduction

The heart is made up of a variety of cells, including cardiomyocytes and non-cardiomyocytes (fibroblasts, smooth muscle cells, endothelial cells, and immune cells etc.,). These cells communicate with each other in both physiological and pathological conditions through direct cell-cell interaction and paracrine signaling. Fibroblasts, the major component of connective tissue, produce the extracellular matrix (ECM) scaffold that organizes different cellular components of the heart (1). Endothelial cells (ECs) are located on the inner surface of blood vessels and lymphatics, controlling vasomotor tension and regulating angiogenesis (2, 3). Resident and recruited immune cells regulate cardiac microenvironmental homeostasis and inflammation during maladaptive remodeling (4). Pathological conditions, such as hypertension or myocardial infarction (MI), induce maladaptive reactions in cardiomyocytes and non-cardiomyocytes, leading to the deterioration of cardiac function and eventually heart failure. However, molecular and cellular mechanisms of cardiac remodeling have not been fully understood.

Other than linear splicing, the sequence of primary transcripts from gene loci is also found to be processed by back-splicing to generate circular RNAs (circRNAs). CircRNAs can be classified as reverse spliced exons (5–7) or intron-derived RNAs (8, 9). Back-splicing is generally thought as a rare event, although, mammalian circRNAs have been reported decades ago (7). The recent deep sequencing data showed evidences that an unexpectedly large number of circRNAs are in fact expressed (5, 6). And thousands of circRNAs have been identified from different cells and tissues (10–13). More importantly, emerging evidences indicated that circRNAs regulate different cellular behaviors, including proliferation, differentiation, apoptosis, and migration (12, 14).

Early report demonstrated the altered expression of circRNAs in human failing heart (15), indicating the participation of circRNAs in the regulation of the pathogenesis of cardiac diseases. Recent studies have further shown that circRNAs are involved in a variety of cardiovascular diseases, including cardiac remodeling, by regulating the pathophysiology of cardiomyocytes, fibroblasts, endothelial cells, and immune cells (16–19). However, the underlying mechanisms of circRNAs' regulatory functions are not fully understood. A more comprehensive understanding of circRNAs will promote the development of circRNA-based diagnosis and therapeutic interventions in cardiovascular disease. In this review, we will focus on the nature of circRNA and how these circular molecules regulate the pathogenesis of cardiac remodeling.

## The Identification, Processing, and Characterization of circRNAs

### Identification of circRNA

In 1976, Sanger used the term “circRNA” for the first time to describe viroids, which was his identification of a single stranded, covalently closed RNA molecules that are infectious (20). Early detection of circRNA was rare and its function was poorly understood. They were believed as by-products of linear RNA and thought as “junk RNA” (21). Three decades ago, circRNAs were accidentally discovered in mammals (22, 23). More and more circRNAs have recently been identified by taking the advantage of the breakthrough of high-throughput sequencing, and the biological functions of these emerging molecules have been investigated and uncovered rapidly (6). circRNAs are currently considered as a special type of non-coding RNAs, although, some studies have suggested that circRNAs may have protein-coding capability *in vivo*.

### General Characteristics of circRNAs

Most circRNAs are exonic and have some important characteristics: (I) CircRNAs are expressed in large quantities in many species, from plants to mammals (5). Multiple circRNA isoforms are often processed from a single host gene by selective splicing. Notably, more than 100 circRNA isoforms of the Ryanodine receptor 2 (RyR2) gene are expressed in human hearts (24). (II) CircRNAs are usually expressed in a cell type- and/or developmental stage-specific manner (6, 25, 26). The expression profiles of circRNAs are different in four stages of cardiac differentiation: undifferentiated stage, mesoderm stage, cardiac progenitor cell stage, and final cardiomyocyte stage (26). (III) CircRNAs are not easily degraded by RNA exonuclease because of their covalently closed circular structure. CircRNAs are more stable and have a longer half-lives than linear RNAs (27, 28). These features of circRNAs make these molecules potential candidates for disease diagnosis and prognosis biomarkers, especially the presence of circRNAs in plasma.

### Categorization of circRNA

CircRNAs can be divided into three subtypes according to the mode of biogenesis: circRNA, Exon-intron circRNAs (EIciRNA), and ciRNAs. Most circRNAs are derived from exons in linear transcripts, lacking introns, and mainly present in the cytoplasm. In contrast, ciRNAs lack exon sequences, are present in the nucleus and have no obvious enrichment of miRNA binding sites (8, 29). EIciRNA sequences contain exons and introns, which are mainly located in the nucleus and form a protein-RNA complex with U1 snRNP and Polymerase II to regulate the transcription of their parent genes (30, 31). CircRNAs can also be classified as intragenic circRNAs and intergenic circRNAs based on the position of circRNA-originated locus in the genome.

### Mechanisms of circRNA Formation

**Figure 1:**
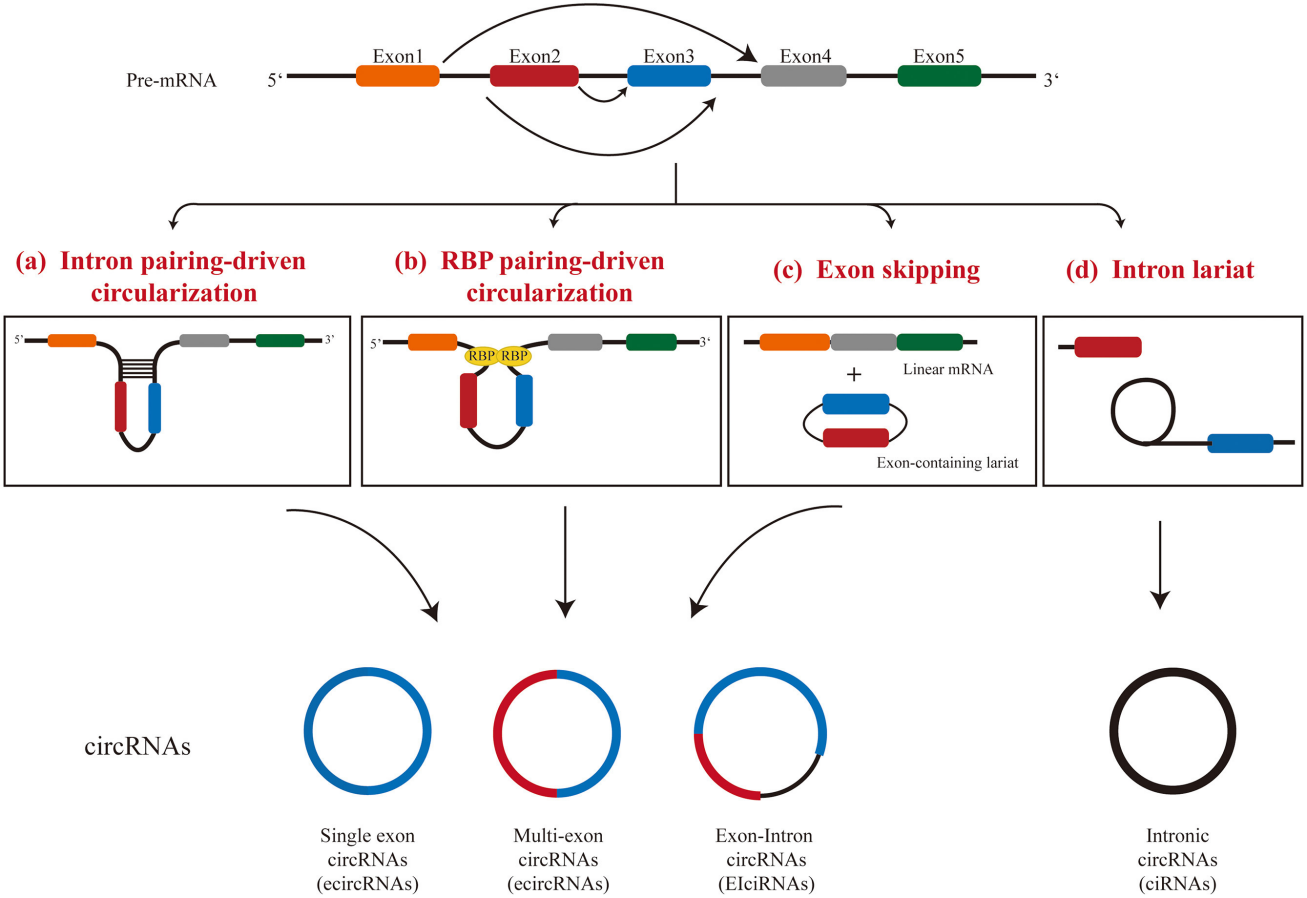
Mechanisms of circRNA formation.

CircRNAs are produced by a unique splicing mechanism called backsplicing (5, 32). Classical splicing events include a typical donor (GU) at the 5 “end of the intron and an receptor (AG) at the 3” end of the intron (33). The circRNAs are formed because the splicing does not finish in the linear manner between intronic donor splicing site next to an exon to the receptor splicing site before the downstream exon, but to the receptor splicing site before the upstream exon. This process produces a covalently closed RNA molecule with or without exons. Three models have been proposed for the formation of circRNAs: (I) intron pairing-driven circularization, (II) RNA binding protein-driven circularization, and (III) lariat-driven circularization (Figure 1).

#### Intron Pairing-Driven Circularization

Compared with linear splicing, the reverse complementary sequences between introns bracketing circRNAs were significantly more abundant (9). In intron-pairing-driven circularization, cis-acting elements, hairpin structures, or complementary sequences located in the flanking introns of an exon are often used for direct base pairing (5, 9, 34). The minimal intron region required for circRNA circularization has been identified (35): even if the intron is <100 nucleotides and contains a typical splicing site sequence and a short reverse repeat sequence, it is sufficient for exon cyclization. This process appears to be more complicated than canonical base pairing, since not all reverse repeats lead to exon cyclization. It is worth noting that multiple exon cyclization events can occur in one gene locus, and exon cyclization efficiency can be modulated by RNA pairing within flanking introns or by competition for RNA pairing within a single intron (36). Alternative formation of reverse repeats of introns, such as repeated ALU pairs, and the competition between them often result in alternative circularization, which leads to the occurrence of producing multiple circRNA transcripts from a gene locus (36). However, how base pairing between introns affects the assembly of the spliceosome during back-splicing is still not fully understood and warrants further investigation.

#### RBP-Driven Circularization

The protein-protein interaction between RNA binding proteins (RBPs) makes the splicing sites of pre-mRNA come closer, which further facilitates the spliceosome to participate in the back-splicing reaction. Multiple RBPs have been shown to regulate the generation of circRNAs. Both the RNA-binding motif protein 20 (RBM20) (37) and splicing factor muscleblind (MBL) (38) were shown to increase the generation of circRNAs by binding to specific intron motifs. RBM20 was identified as an important splicing factor in the heart with the function of regulating the formation of circRNAs in TTN gene locus (37).

A further study suggested that the RBP-driven circularization and the intron-pairing-driven circularization may work together to regulate the formation of circRNAs (39). Intronic repeats in flanking introns are believed to provide an opportunity for RBM20 to facilitate the circularization event. After that, a subset of proteins are recruited, which further regulate the formation of circRNA by modulating the activity of spliceosomes. Each gene locus may require a different set of protein factors for the generation of multiple circRNAs.

#### Lariat-Driven Circularization

Interestingly, a circRNA can also be produced during linear splicing by lariat-driven circularization, in which circRNAs may be generated during exon-skipping events (40) or intron removal in pre-mRNA splicing (8). TTN gene is an example of generating circRNAs through exon-skipping events with more than 80 circRNAs generated through this mechanism in the heart (37). Lariat RNAs are the intermediate product of splicing of pre-mRNA. Under normal circumstances, lariat RNAs released in canonical splicing undergo debranching at the 2′-5′ phosphodiester bond, and are then degraded by exonucleases (41). However, the specific structure of 7 nt GU-rich near the 5′ splice site and 11 nt C-rich near the branching site of lariats prevent the debranching event, and therefore, these RNA molecules remain circular (8, 42). These type of circular RNAs become mature after the 3′ tail of the lariat is degraded up to the branching point (43).

## The Regulatory Mechanisms of circRNAs

**Figure 2:**
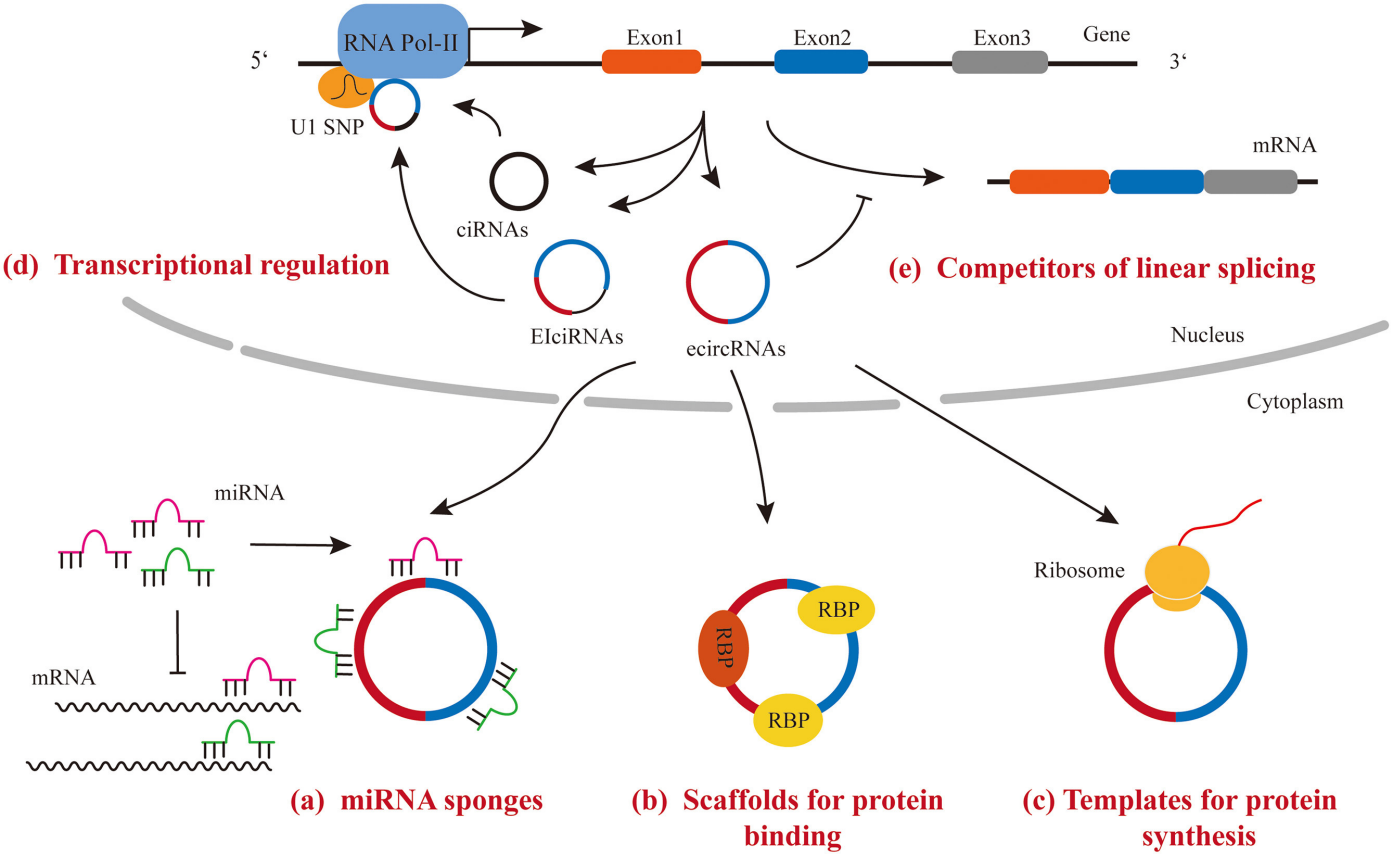
Molecular mechanisms of regulatory function of circRNAs.

Accumulating data have shown that circRNAs exert their regulatory function through the following mechanisms: (1) functioning as miRNA sponges to sequester miRNAs and de-repress their targets; (2) functioning as scaffolds to bind RNA binding proteins and regulate the activity of downstream signaling; (3) binding to snRNP and polymerase II to regulate transcriptional activity; (4) functioning as competitors for parental gene splicing and expression; (5) functioning as templates for protein synthesis (Figure 2).

### MicroRNA Sponge

MiRNAs repress gene expression post-transcriptionally by binding to the 3′ UTR of target mRNAs. CircRNAs have been demonstrated to possess multiple miRNA binding sites by both computational prediction and experimental assays. The interaction between circRNA and miRNA leads to miRNA retention and then lowering their bioactivity, which is referred to the “sponge effect.” For example, a CDR1 locus-derived circRNA, CDR1as, has 63 highly conserved miR-7 binding sites (6, 44). Since no linear transcript of CDR1as were detected, the knockout strategy is simple and the removal of DNA sequence for circRNA from the genome will not affect the expression of any linear transcript from the same DNA locus. The high expression level of CDR1as and the presence of large amount miR-7 binding sites per molecules makes the circRNA a competitive inhibitor of endogenous miR-7.

### Scaffold for Protein Interaction

Some circRNAs possess protein binding motifs. Therefore, these circRNAs interact with selected proteins and regulate their activity or localization. For example, the interaction between circPABPN1 and HuR prevents HuR from binding to PABPN1 mRNA and reduces its translation (45). In another example, circMBL, a circRNA derived from MBL gene locus and containing the conserved binding site of MBL, binds to MBL and regulates the splicing of its own pre-mRNA (38). However, computational analysis predicted that the density of RBP binding sites is lower in circRNAs than in 3′ UTR regions of protein-coding genes (46).

### Transcriptional Regulation

Most circRNAs are presented in the cytoplasm and act as either miRNA sponges or scaffolds. However, ciRNA and EIciRNA, such as circEIF3J and circPAIP2, remain in the nucleus and interact with U1 snRNA and RNA polymerase II complex to enhance the transcriptional activity of their parent gene (31). However, the underlying mechanism of the regulatory function of EIciRNA remains unclear.

### Competitors of Linear Splicing and Gene Expression

The process of circRNA formation also affects the expression of host gene. Some evidences indicated that circRNA formation competes strongly with the linear splicing of pre-mRNA, and therefore, regulates host gene expression (38, 47). For example, an increase in linear splicing efficiency in Drosophila S2 cells led to a decrease in circRNA expression (38). In another example, a decrease in spliceosome components in drosophila cells resulted in an increase in circRNA levels and a decrease in its associated linear mRNA expression (47). Since linear- and back-splicing use the same typical splicing receptor and donor, it is not surprising that the level of circRNAs is negatively associated with the level of their linear mRNA isoforms.

### Templates for Protein Synthesis

Although, circRNAs were first identified as non-coding RNAs, some of these circular RNA molecules were found to have the protein/peptide coding capability. Given that circRNAs are mostly localized in the cytoplasm and contain protein-coding exons, people wonder whether they can be loaded into ribosomes and serve as a template for protein/peptide synthesis. Interestingly, studies showed the initiation of translation of circRNAs can occur either at the internal ribosomal entry site (IRES) or at nucleotides with m6A modification in 5′ untranslated region (UTR) (48, 49), although, they lack the cap-dependent translation elements. So far, only a few endogenous circRNAs, such as circFBXW7, circMBL, and circ-ZnF609, have been shown to possess the effective open reading frame for protein/peptide translation (50–55). The function of most circRNA-derived peptides is unknown. It is worthy to note that circRNA-derived peptides were found to be expressed under different stress conditions, such as the translation of circ-ZnF609 in response to heat shock (50, 51). Although, translation of circRNAs does not appear to be a common function of circRNAs, the next important task in this field is to determine the regulatory function of circRNA-derived proteins/peptides.

## Participation of circRNAs in the Pathogenesis of Cardiac Remodeling

Due to the limited regenerative capacity of myocardial tissue, the heart undergoes extensive remodeling to compensate the loss of cells or response to stress. During remodeling, hypertrophic growth and limited proliferation occur in cardiomyocytes. In addition, non-cardiomyocytes such as cardiac fibroblasts, endothelial cells, smooth muscle cells and immune cells, are all shown to actively participate in this disease progress. For example, the dying cardiomyocytes secret cytokines to activate the proliferation and differentiation of cardiac fibroblast, and to recruit immune cells to clean up the dead cardiomyocytes; ischemic and oxidative stress also trigger the proliferation of endothelial cells and angiogenesis for re-establishment of blood supply. Different cell types are tightly act together and communicate with each other either through cell-cell junction or cytokines, or even through exosomes during this process.

**Figure 3:**
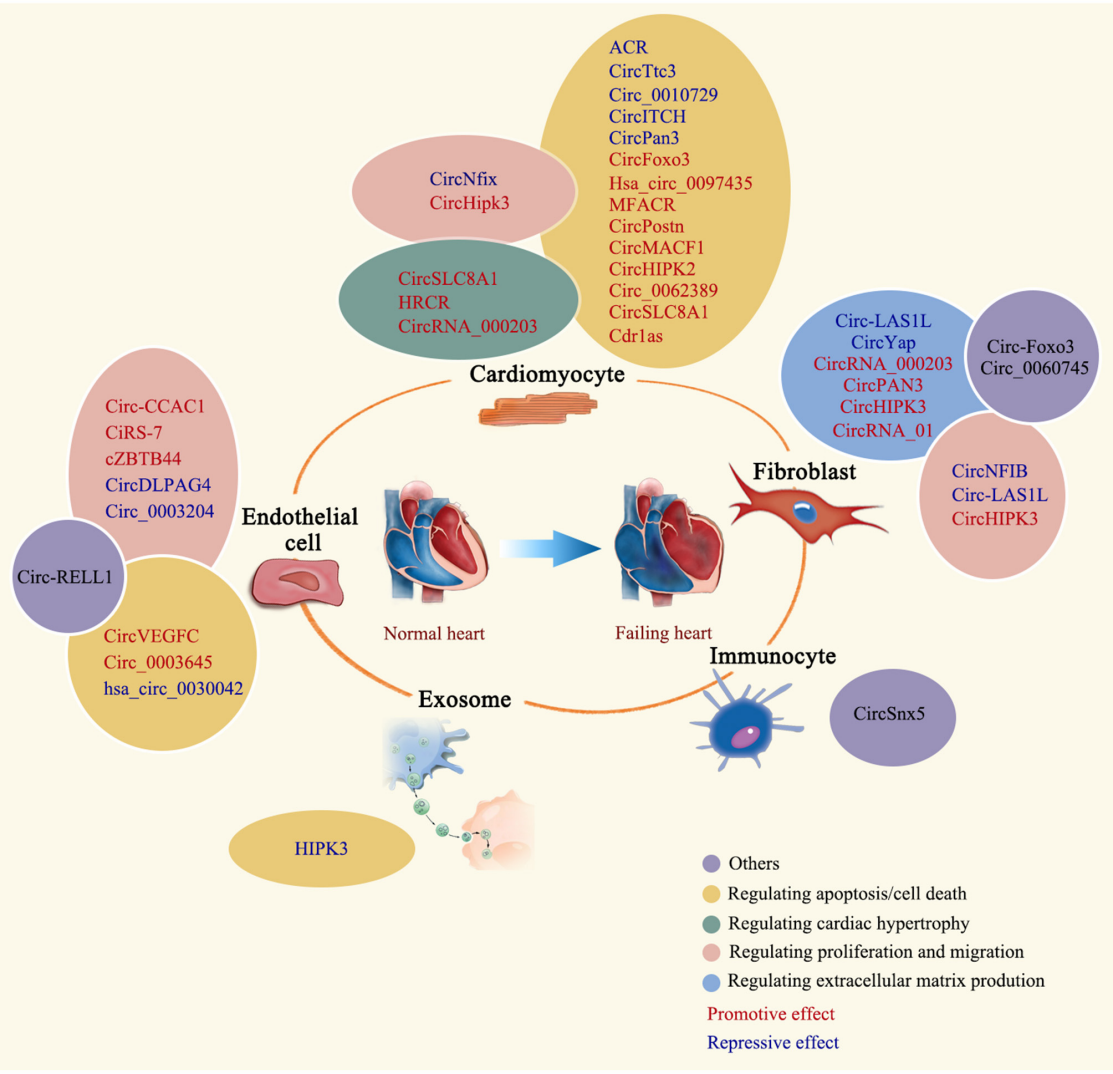
Regulatory roles of circRNAs in cardiac remodeling. The regulatory role of summarized circRNAs is indicated by colored ovals. CircRNAs are labeled in different colors to indicate the promotive or repressive function.

More and more evidences demonstrated that non-coding RNAs constitute a regulatory network in almost all forms of human diseases, including cardiac remodeling and heart failure. These RNA molecules incorporate into the known protein regulatory network to orchestrate a highly complicated gene regulatory network in human diseases. Cracking the “code” of this network will provide us a roadmap to fully understand mechanisms beneath disease phenotype we observed, and eventually lead us to a better and more effective therapy. Previous studies have demonstrated that some non-coding RNAs, such as microRNAs and long non-coding RNAs, have altered expression and play important regulatory roles in cardiac remodeling (14, 56, 57). Due to the different structure of circular RNAs, this type of non-coding RNAs have not been broadly studied until recently (58). Studies showed that circular RNAs are widely presented in different mammalian cell types (5). Growing evidence shows that circRNAs play important roles in cell proliferation, apoptosis, migration, and differentiation (43, 50, 59). Importantly, RNA-sequencing data showed that a subset of circular RNAs are dysregulated in diseased heart (37), supporting the idea of circular RNAs possessing regulatory functions in cardiac remodeling. Here, we systematically review the recent study progress of circRNAs in cardiac remodeling (Table 1 and Figure 3) and discuss the function of some representative circRNAs in cardiomyocytes, fibroblasts, endothelial cells, immune cells, and exosomes in detail.

**Table 1:** A list of reported regulatory circRNAs in cardiac remodeling.

**CircRNAs**	**Host gene**	**Target**	**Mechanism**	**Function**	**References**
**CircRNAs in cardiomyocytes**					
HRCR	HRCR	miR-223	miRNA sponge	Promoting cardiac hypertrophy and heart failure	(16)
CircSLC8A1	NCX	miR-133a-3p	miRNA sponge	Promoting cardiomyocytes apoptosis and hypertrophy	(60, 61)
Cdr1as	Cdr1as	miR-7	miRNA sponge	Promoting cardiomyocyte apoptosis	(62)
CircNfix	Nfix	miR-214/interaction between Ybx1 with Nedd4l	miRNA sponge/Scaffolds for protein interaction	Inhibiting cardiomyocyte proliferation and angiogenesis	(63)
ACR	ACR	Dnmt3B	Transcriptional regulation	Inhibiting autophagy and cell death	(64)
CircHipk3	Hipk3	N1ICD, miR-185-3p, miR-17-3p	Scaffold for protein interaction/miRNA sponge	Promoting cardiomyocyte proliferation	(65)
CircHIPK2	Hipk2	miR-485-5p	miRNA sponge	Promoting autophagy and apoptosis	(66)
CircITCH (hsa_circ_0001141)	ITCH	miR-330-5p	miRNA sponge	Inhibiting cardiomyocyte apoptosis	(67)
CircPan3	Pan3	Undetermined	Undetermined	Inhibiting cardiomyocyte apoptosis	(68)
CircFoxo3	Foxo3	Foxo3	Scaffold for protein interaction	Promoting cell apoptosis/death	(69)
CircRNA_000203	Myo9a	miR-26b-5p, miR-140-3p	miRNA sponge	Promoting cardiac hypertrophy	(70)
CircTtc3	Ttc3	miR-15b-5p	miRNA sponge	Inhibiting ATP depletion and apoptotic death	(71)
Circ_0010729	Undetermined	miR-27a-3p, miR-145-5p, miR-370-3p	miRNA sponge	Inhibiting apoptosis and glycolysis	(72–74)
MFACR	MFACR	miR-652-3p	miRNA sponge	Promoting mitochondrial fission and the apoptosis of cardiomyocytes	(75)
CircMACF1	MACF	miR-500b-5p	miRNA sponge	promoting cardiomyocyte apoptosis	(76)
Hsa_circ_0097435	Undetermined	Undetermined	Undetermined	Promoting cardiomyocyte apoptosis	(77)
Circ_0062389	PI4KA	Undetermined	Undetermined	Promoting cardiomyocyte apoptosis	(78, 79)
CircPostn	Postn	miR-96-5p	miRNA sponge	Promoting cardiomyocyte apoptosis	(80)
**CircRNAs in cardiac fibroblasts**					
CircRNA_000203	Myo9a	miR-26b-5p	miRNA sponge	Promoting fibrotic phenotype of CFs	(81)
Circ_0060745	Undetermined	Undetermined	Undetermined	Increasing myocardial infarct size and worsening cardiac functions after AMI and contributes to activation of NF-κB under hypoxia	(82)
CircNFIB	Nfib	miR-433	miRNA sponge	Inhibiting CFs proliferation	(17)
Circ_LAS1L	LAS1L	miR-125b	miRNA sponge	Inhibiting the activation, proliferation, migration and promotes apoptosis of CFs	(83)
CircPAN3	PAN3	miR-221	miRNA sponge	Promoting fibrotic phenotype of CFs and activation of autophagy	(84)
CircHIPK3	HIPK3	miR-29b-3p	miRNA sponge	Promoting proliferation, migration of CFs and development of cardiac fibrosis	(85, 86)
		miR-152-3p	miRNA sponge	Promoting proliferation, migration and phenotypic transformation of CFs	(87)
CircRNA_010567	Undetermined	miR-141	miRNA sponge	Promoting fibrotic phenotype of CFs	(88)
CircYap	YAP	TPM4 and ACTG	Scaffold for protein interaction	Inhibiting fibrotic phenotype and migration of CFs	(89)
Circ-Foxo3	Foxo3	ID-1, E2F1, FAK and HIF1α	Scaffold for protein interaction	Promoting senescence of CFs	(90)
**CircRNAs in endothelial cells**					
Circ-CCAC1	ERBB2	EZH2	Scaffold for protein interaction	Disrupting endothelial barrier integrity and promoting angiogenesis	(91)
Circ_0003204	USP36	miR-370-3p	miRNA sponge	Inhibiting proliferation, migration and tube formation of endothelial cell	(92)
CircDLPAG4	DLGAP4	miR-143	miRNA sponge	Inhibiting endothelial cell migration, without affecting cell viability, and apoptosis	(93)
CircVEGFC	VEGFC	miR-338-3p	miRNA sponge	Promoting vascular endothelial cells apoptosis	(94)
Circ-RELL1	RELL1	miR-6873-3p	miRNA sponge	Promoting inflammation in ECs	(95)
CiRS-7	LINC00632	miR-26a-5p	miRNA sponge	Promoting tube formation in microvascular endothelial cells	(96)
Circ_0003645	chr16:19656207- 19663412	Undetermined	Undetermined	Promoting endothelial cell inflammation and apoptosis after silencing	(97)
CZBTB44	chro11: 130130750-130131824	miR-578	miRNA sponge	Promoting cell viability, proliferation, migration and tube formation	(98)
Hsa_circ_0030042	FOXO1	eIF4A3	Scaffold for protein interaction	Inhibiting abnormal autophagy	(99)
**CircRNAs in immunocytes**					
CircSnx5	Snx5	miR-544/SOCS1	miRNA sponge	Inducing immunological tolerance	(19)
**CircRNAs in exosomes**					
CircHIPK3	HIPK3	miR-29a	miRNA sponge	Inhibiting endothelial cell apoptosis	(100)

### Circular RNAs in Cardiomyocyte

As the main contractile cells in the beating heart, the alteration of cardiomyocytes is the central of cardiac remodeling in various disease status. In adult heart, cardiomyocytes occupied around 75% of left ventricular volume (101). Cardiomyocytes undergo hypertrophic growth, apoptosis/necrosis, and limited proliferation during cardiac remodeling. Non-coding RNAs, such as microRNAs and long non-coding RNAs, have been demonstrated to regulate the pathophysiology of cardiomyocytes in diseased heart (102). Here, we discussed some emerging examples of circRNAs in cardiomyocytes during cardiac remodeling.

#### HRCR

HRCR was identified as the first circRNA regulating cardiac hypertrophy (16). The expression of circRNA HRCR was shown substantially decreased in mice in response to ISO or TAC treatment. HRCR has a protective function on cardiac hypertrophy and heart failure. Forced expression of HRCR mediated with adenoviral constructs in mouse heart decreases cardiomyocyte hypertrophic growth, interstitial fibrosis and preserves the cardiac function upon ISO treatment. Mechanistically, six target sites for miR-223 were identified in HRCR. HRCR sequesters and decreases the activity of miR-223, and upregulates the expression of target of miR-223, apoptosis repressor with CARD domain (ARC), which is a known regulator of cardiomyocyte hypertrophy and apoptosis (16).

#### CircSLC8A1

CircSLC8A1 (also named CircNCX1, or named circSlc8a1-1 in mouse), which is enriched in cardiomyocytes, was identified as the most abundant circRNA in human and mouse heart (103), whose host gene encodes the protein of sodium-calcium exchanger (NCX). CircSLC8A1 is generated from the 2nd exon of the host gene SLC8A1. Although, the expression of CircSLC8A1 remains unaltered under some disease conditions such as cardiac hypertrophy in mouse and failing heart in human, it has been confirmed to be involved in regulation of hypertrophic growth of cardiomyocytes as an endogenous sponge for miR-133a (24, 37, 60, 104). Of note, inhibition of circSLC8A1 promotes TAC-induced hypertrophy and HF in mouse (60). Cardiac-specific overexpression of circSlc8a1 *in vivo* mediated by the AAV9 increases heart weight and results in cardiac dilatation. Different from the expression in hypertrophy, the expression of circSLC8A1 have been demonstrated abnormally increased in dilated cardiomyopathy (105, 106), and upregulated in ischemic rat cardiac cells and mouse heart (61). Through a similar mechanism of acting as miR-133a-3p sponge, the NCX-derived circRNA increased the levels of CDIP1, a target for miR-133a-3p, which promotes cardiomyocyte apoptosis. Therefore, circSLC8A1 could exert different regulatory function in cardiomyocytes depending on the type of stress. Interestingly, circSLC8A1 interacts with the mouse ribosome or rat Argonaute 2 protein, which indicates circSLC8A1 is likely involved in the regulation of mRNA translation (106). Interesting, the expression level of circSLC8A1 is increased and positively correlated with the expression of CK-MB in the pericardial fluid of acute ischemic heart disease patients, which shows the potential of using this circRNA as an auxiliary diagnostic marker for clinical acute coronary syndromes (107).

#### CircNfix

CircNfix was identified as a super enhancer-associated circRNA by an integrated analysis with RNA-seq data and super enhancer catalogs (63). CircNfix was shown to regulate cardiomyocyte proliferation and angiogenesis. *In vivo* knockdown of circNfix mediated by cTNT-driven shRNA expression through AAV9 viral delivery system promotes cardiomyocyte proliferation evidenced by the increased the expression of the proliferation markers and the total cardiomyocyte number in infarcted mouse hearts, which leads to an improved cardiac function after MI. Although, CircNfix functions as a miRNA sponge to decrease the promotive function of miR-214 on cardiomyocyte proliferation, unexpectedly, it also promotes Ybx1 degradation through ubiquitination by enhancing the stability of the interaction between Ybx1 with Nedd4l, an E3 ubiquitin ligase. This observation indicates that CircNfix also functions as a scaffold for protein docking. Since transcriptional factor Ybx1 activates the expression of Ccna2 and Ccnb1 (108), CircNfix further decreases the proliferation of cardiomyocyte during myocardial infarction.

#### ACR

A recent study demonstrated that a circRNA ARC (autophagy-related circular RNA) plays an important role in cardiomyocyte autophagy (64). The expression of circRNA ACR is markedly decreased after the heart subjected to ischemia/reperfusion. ACR attenuates the increased autophagy level upon ischemia/reperfusion injury and plays a protective role in cardiomyocytes *in vivo*. Mice with overexpression of ACR in the heart had a less cardiomyocyte death in ventricular tissue and a smaller infarction. Mechanistically, instead of a miRNA sponge, ACR acts as a regulator of chromatin modification by binding to Dnmt3B and inhibiting Dnmt3B-mediated DNA methylation of Pink1 promoter. Since Pink1 targets and phosphorylates FAM65B, which was shown to have a regulatory role in autophagy, ARC mediates cardiomyocyte autophagy through a Dnmt3B/Pink1/FAM65B signaling cascade.

#### CircHipk3

Similar to ACR, some of circRNAs have been demonstrated to serve as a signaling regulator by binding with protein in cardiomyocytes. A recent study found an increased expression level of circHipk3 in the fetal or neonatal mouse heart (65). AAV9-mediated overexpression of circHipk3 attenuated cardiac dysfunction and fibrosis in a mouse model of myocardial infarction. CircHipk3 was shown to interact with N1ICD protein to increase N1ICD acetylation level and stability, which was partially responsible for the beneficial effect of circHipk3 in cardiomyocytes. On the contrary, silencing circHIPK3 has a protective effect in a variety of heart diseases. The hypertrophic growth of cardiomyocytes was markedly inhibited by the knockdown of circHIPK3 in a TAC-induced cardiac hypertrophy model (109). Since CircHIPK3 is a sponge of miR-185-3p, decreased level of CircHIPK3 in the knockdown increases the inhibitory effect of miR-185-3p on CaSR. In addition, knockdown of circHIPK3 benefits the heart after myocardial infarction through a circ-HIPK3/miR-17-3p/ADCY6 signaling cascade in cardiomyocytes (110). While in a model of LPS-induced myocarditis, knockdown of circHIPK3 significantly represses cardiomyocyte apoptosis and alleviates oxidative stress and inflammation in cardiac tissue (111). Interestingly, it was reported that circHIPK2, originated from the second exon of another HIPK family member HIPK2, facilitated autophagy in H2O2-caused myocardial injury *via* sponging miR-485-5p and de-repressing miR-485-5p target, ATG101 (66, 112).

#### Other circRNAs in Cardiomyocytes

Doxorubicin is widely used in tumor chemotherapy, however, with the dose-dependent cardiotoxicity. Doxorubicin-induced cardiotoxicity involves many molecular mechanisms, including induction of reactive oxygen species (113), inhibition of the activity of topoisomerase II (114), interruption of calcium homeostasis, induction of mitochondrial dysfunction, and destruction of sarcomere function (115). Recent studies showed that circRNAs are involved in doxorubicin-induced cardiomyopathy. A previous study identified 356 differentially expressed circRNAs in doxorubicin-treated human hearts (67). CircITCH, a circRNA highly conserved between human and mice, was significantly downregulated in DOX-induced cardiomyopathy. AAV9-mediated overexpression of circITCH ameliorates oxidative stress and DNA damage, cell death, contractile dysfunction, and calcium handling defects in DOX-induced cardiomyopathy in a mouse model by acting as a miR-330-5p sponge to de-repress the expression of SIRT6, BIRC5, and ATP2A2. In another recent study, overexpression of circPan3 was shown to attenuate DOX-induced cardiomyocyte apoptosis with unknown mechanism (68). In another example, circ-Foxo3 interacts with the anti-senescence proteins ID1 and E2F1, and anti-stress proteins FAK and HIF1α to prevent their nuclear translocation for transcription (90). Therefore, silencing of circ-Foxo3 relieved the cardiac injury induced by doxorubicin. In addition, circFoxo3 levels were significantly higher in I/R injury resulted from 24 h of cold storage and reperfusion in heart transplantation (69). *In vivo* and *in vitro* experiments demonstrates that knockdown of circFoxo3 improves heart graft function and reduced cell apoptosis/death and mitochondrial damage.

### Circular RNAs in the Activation of Cardiac Fibroblasts

Cardiac fibrosis, generally referred to an aberrant accumulation of extracellular matrix (ECM) proteins in the interstitial space of heart tissue, is closely associated with pathological cardiac remodeling. It manifests as deposition of scar, increasing stiffness, decreasing contraction and impaired heart function, which ultimately resulting in heart failure (116). Although, cardiac fibrosis is a complex process and involves many types of cells in the heart, such as cardiomyocytes, fibroblasts, lymphocytes, and pericytes (117–119), extensive studies have proved that cardiac fibroblasts (CFs) play a pivotal role in this process (120). When suffered cardiac injury, the proliferation and migration of CFs are increased. Moreover, cytokines, especially the transforming growth factor-β (TGF-β), and growth factors are also secreted, which contributes to fibroblast activation and ultimately transform CFs into myofibroblasts (121). In this situation, myofibroblasts begin to express α-smooth muscle actin (α-SMA) and enhance the secretion of ECM proteins, such as collagen type I and collagen type III, which results in the formation of scar and eventually leads to cardiac fibrosis (122). Recent studies have shown circRNAs actively participate in the pathogenesis of cardiac fibrosis, especially in the activation of cardiac fibroblasts.

#### CircRNA_000203

CircRNA_000203, derived from Myo9a, is upregulated in the myocardium of diabetic mouse and Ang-II-treated cardiac fibroblasts. Overexpression of circRNA_000203 in mouse CFs *in vitro* increased the expression of Col1a2, Col3a1, and α-SMA, indicating the activation of CFs and the accumulation of ECM. Moreover, RNA pull-down confirmed that circRNA_000203 is a sponge of miR-26b-5p. The targeting of CTGF and Col1a2 by miR-26b was experimentally confirmed, which was interfered by the overexpression of circRNA_000203 (81). Interestingly, circRNA_000203 is also upregulated in Ang-II-treated cardiomyocytes. Forced expression of circRNA_000203 promotes the hypertrophic growth of cardiomyocytes and transgenic mice with cardiac-specific overexpression of circRNA_000203 have an advanced phenotype in a model of Ang-II-induced cardiac hypertrophy (70).

#### CircNFIB

CircNFIB (mmu_circ_0011794), generated from the exon regions of Nfib, was identified as a candidate circRNA to sponge miR-433, a miRNA promoting cardiac fibrosis (123). CircNFIB has a decreased expression in both 3-week post-MI mice hearts and TGF-β-treated CFs. The proliferation of CFs, induced by TGF-β treatment, was significantly inhibited by the overexpression of circNFIB *in vitro* (17). Mechanistically, circNFIB de-represses AZIN1 and JNK1, which are targeted by miR-433. Overexpression of circNFIB increased the expression of AZIN1 and JNK1 and impaired the activation p38/ERK/Smad3, thus confirming the pivotal role of circNFIB as a competing endogenous RNA (ceRNA) in cardiac fibrosis. However, the role of circNFIB *in vivo* in cardiac remodeling is still unclear and requires for further investigation.

#### Circ_LAS1L

CircRNAs have been found to be involved in acute myocardial infarction (AMI) in recent years (124). Bie et al. (125) demonstrated the crucial function of miR-125b/SFRP5 axis in CF growth and activation in previous report. Further, analysis showed that miR-125b targets circ_LAS1L with two binding sites, which was confirmed by RIP and RNA Pull-down. The expression level of circ_LAS1L is significantly downregulated while miR-125b expression is increased in AMI patients. Forced expression of circ_LAS1L upregulates the expression of SFRP5 and downregulates the expression of α-SMA, collagen I, and collagen III in CFs. Interestingly, gain function of miR-125b together with overexpression of circ_LAS1L appears not to modulate CFs proliferation, apoptosis, and migration. However, SFRP5 siRNA, instead of miR-125b mimics, bypassed the counter effect of circ_LAS1L on CF proliferation and migration, indicating that circ_LAS1L functions as a sponge of miR-125b to modulate CFs proliferation and migration *in vitro* (83). Whether circ_LAS1L has a repressive function on cardiac fibrosis upon cardiac injury *in vivo* warrant investigation in the future.

#### CircRNA_PAN3

CircPAN3, a circRNA generated from the PAN3 locus, has been found to maintain the self-renewal of intestinal stem cells (126), to modulate drug resistance in acute myeloid leukemia (AML) (127, 128), and to recede myocardial ischaemia/reperfusion injury (129). Recently, Li et al. reported circPAN3 as a new profibrotic factor in cardiac fibrosis (84). The expression of circPAN3 increases significantly in fibrotic regions of rat heart induced by myocardial infarction (MI). Silencing of circPAN3 in MI hearts reduces the level of fibrosis, including decreased expression of fibrotic markers, and inhibits cardiac myocyte apoptosis and autophagy. Consistently, knockdown of circPAN3 represses TGF-β induced proliferation, migration and autophagy of CFs *in vitro*. In the molecular level, circPAN3 was demonstrated to interact with miR-221 and sequester miR-221 from regulate its targets, FoxO3, as a sponge. Gain function of miR-221 decreases the expression of FoxO3 and ATG7, two known targets for miR-221 and have been proved crucial in autophagy in previous studies (130). These data indicate that circPAN3 promotes fibrosis *via* miR-221/FoxO3/ATG7 cascade-mediated autophagy.

#### Circ_0060745

A recent study reported that circ_0060745 expression level in CFs is increased dramatically in the myocardium of AMI mice (82). Knockdown and overexpression of circ_0060745 improved and deteriorated the cardiac function, respectively. In addition, the silencing of circ_0060745 leads to less cell apoptosis in the infarcted areas while circ_0060745 overexpression had the opposite effect. Further, analysis found that the expression of inflammatory cytokines, including IL-6, IL-12, IL-1β, and TNF-α, are decreased upon knockdown of circ_0060745, which could suppress peritoneal macrophage migration. The downregulation of the inflammatory cytokines is induced by the inhibition of NF-κB activation in the circ_0060745 knockdown.

#### CircHIPK3

CircHIPK3 (mmu_circ_0001052), originated from exon 2 of HIPK3, has been reported to play an important role in cancers (131). Recent studies suggested it also regulates cardiac fibrosis *via* different signaling cascades. Ni et al. found circHIPK3 promoted CF proliferation, migration and activation by modulating the activity of a known fibrosis-related microRNA, miR-29b-3p, through sponging (85). Similarly, another study showed circHIPK3 induced cardiac fibrosis through a circHIPK3/miR-29b-3p/Col1a1/Col3a1 signaling cascade in mouse diabetic cardiomyopathy model (86). Interestingly, circHIPK3 modulates CFs function in a hypoxia condition in a similar sponge manner, but through a different signaling cascade, the circHIPK3/miR-152-3p/TGF-β2 axis (87). Therefore, circHIPK3 is likely to be an important upstream node of the miRNA-mediated posttranscriptional gene regulation network in cardiac fibrosis.

#### CircYap

CircYap hsa_circ_0002320, generated from exons 5 and exon 6 of YAP pre-mRNA, is the highest expressed isoforms derived from Yap gene locus in human hearts. The expression level of circYap decreases significantly in hypertrophic patient hearts of patients and in pressure-overloaded mouse hearts. The forced expression of circYap alleviates the declined heart function and increased cardiac fibrosis in a TAC-induced mouse cardiac hypertrophy model. Overexpression of circYap in cardiac fibroblasts (MCF) *in vitro* suppresses the expression of fibrotic markers and migration of cardiac fibroblasts. Mechanistically, circYap interacts with both tropomyosin-4 (TMP4) and gamma-actin (ACTG), and enhances the interaction between TMP4 and ACTG, which subsequently inhibits the actin polymerization and cardiac fibrosis (89).

### Circular RNAs in Endothelial Cells

Endothelial cells (ECs) play a central role in cardiac remodeling, regeneration, as well as angiogenesis in the treatment of cardiovascular diseases (132, 133). Therefore, it is of great significance to identify factors that promoting and inhibiting angiogenesis and their underlying molecular mechanisms. To date, many studies have confirmed that circRNAs are involved in regulating the proliferation, migration, apoptosis, and tubule formation of ECs, which further mediates the dynamics of ECs and regulates angiogenesis.

#### Circ-ZnF609

The level of circ-ZNF609 in peripheral blood leukocytes of coronary artery disease patients is significantly decreased (134). In another study of circRNAs in retinal vascular dysfunction, Circ-ZnF609 was found significantly upregulated under conditions of high glucose and hypoxia stress *in vivo* and *in vitro* (18). Silence of circ-ZnF609 increased endothelial cell migration and tube formation, and protected endothelial cells against oxidative stress and hypoxia stress *in vitro*. Circ-ZnF609 acts as an endogenous miR-615-5p sponge to sequester miR-615-5p and inhibit its function, leading to the increased expression of MEF2A. Overexpression of MEF2A rescues the endothelial cell migration, tube formation, and apoptosis mediated by the silence of circ-ZnF609, which further demonstrates the regulatory mechanism of circ-ZnF609 on ECs *via* circ-ZnF609/miR-615-5p/MEF-2A signaling cascade.

Interestingly, circ-ZnF609 was also identified as a circRNA regulating muscle differentiation in mice and humans, and its expression is altered in Duchenne muscular dystrophy (DMD) myoblasts (50). Circ-ZnF609 specifically controls the proliferation of myoblasts. It was demonstrated that circ-ZnF609 is associated with heavy polysomes and translated into a protein in a splicing-dependent and cap-independent manner, providing an example of protein encoding circRNA in eukaryotes. Whether circ-ZnF609 functions as a miRNA sponge or protein-coding circRNA, or both, or it has a preferable manner depended on cell type, needs to be studied in the future. Furthermore, the role of endothelial circ-ZnF609 in the heart upon ischemia/reperfusion *in vivo* warrants investigation.

#### CircFndc3b

CircFndc3b is differentially expressed in the mouse hearts after myocardial infarction (MI) and in the heart tissues of patients with ischemic cardiomyopathy (135). Overexpression of circFndc3b in cardiac endothelial cells increases the expression of vascular endothelial growth factor-A, enhances angiogenesis, and reduces the apoptosis of cardiomyocytes and endothelial cells. In post-MI hearts, adeno-associated virus-mediated overexpression of circFndc3b reduces myocardial apoptosis, enhances neovascularization, and improves left ventricular function. CircFndc3b interacts with the RNA-binding protein FUS in sarcoma to regulate VEGF expression and signal transduction. These findings highlight the physiological role of circRNA in heart repair and suggest that regulating the expression of CircFndc3b is a potential therapeutic strategy for protecting the heart from myocardial infarction.

#### CZNF292

Hypoxia condition is introduced in the ischemic region of the diseased heart. To identify hypoxia-related circRNAs, endothelial circRNAs were screened from human umbilical vein endothelial cells cultured under normal or hypoxia conditions (136). CZNF292 is one of candidates identified in the screen. *In vitro*, target-specific depletion of CZNF292 with siRNAs inhibits angiogenic germination and spherical germination of endothelial cells, suggesting that CZNF292 has pro-angiogenic function. The overexpression of CZNF292 further confirms its pro-proliferation effect. Interestingly, the circRNA appears not associate with Argonaute, indicating it unlikely functions as a microRNA sponge. These data suggest that endothelial circRNAs could mediate angiogenesis under hypoxia condition with undetermined mechanism. Although, the *in vivo* function of CZNF292 in the heart is still unknown, it is interesting to investigate whetherCZNF292 promotes angiogenesis *in vivo* and benefit the heart after ischemia/reperfusion injury.

### Circular RNAs in the Dynamics of Cardiac Immunocytes

Inflammation and fibrosis are two key factors in cardiac remodeling. Inflammatory response is generally caused by acute cell death, for instance, the sudden loss of cardiomyocytes after myocardial infarction. Necrotic cardiomyocytes crack and release cellular contents, which activates the inflammation reaction for cleaning dead cells and matrix debris (137, 138). During the cardiac inflammatory process, immunocytes accumulate in myocardium, infiltrate surrounding tissue and further regulate inflammatory reaction (139, 140). Among immunocytes, dendritic cells (DCs), derived from bone marrow, are antigen-presenting cells and crucial in immune response. Moreover, cardiac DCs has been reported to have heart-protective effects in acute myocardial infarction (AMI), such as the deletion of DCs in mice deteriorates the cardiac remodeling (141). In addition, cardiac specific tDCs (tolerogenic dendritic cells) can evoke the generation of Tregs, which can promote a macrophage-specific repair program after AMI (142).

Recently, Yu et al. reported a novel DC-expressed circRNA, named circSnx5, has a vital function in maintaining cardiac immune homeostasis. CircSnx5, generated from the snx5 gene locus, represses the maturation of DCs when its expression is upregulated in DCs. Knockdown of circSnx5 results in an inflammatory phenotype of dendritic cells. Mechanistically, circSnx5 sponges miR-544 and de-represses the downstream target of miR-544, the suppressor of cytokine signaling 1 (SOCS1). On the other hand, circSnx5 directly influences the nuclear translocation of PU.1 to regulate the expression of downstream MHC class II, which is critical to DC's function. In addition, the injury and inflammation of cardiac tissue is decreased, and the cardiac function is improved after introducing circSnx5-overexpressing DCs into experimental autoimmune myocarditis (EAM) mice. Thus, all these results confirm that circSnx5 has a protective effect on AMI (19).

### Circular RNA Messenger in Exosomes

It is well-known that exosomes are involved in the intercellular communication. Correct communication between cells has been shown critical in preserving body homeostasis and health (102). Cell-cell communication *via* exosomes is involved in the pathological processes of some chronic diseases such as cancer and heart diseases (143, 144), but the disease progression regulated by circRNAs from the shuttling exosomes in the heart were less studied (145).

A recent study has provided evidence supporting the role of exosomal circRNAs in multiple physiological processes including the regulation of the heart function. Wang et al. found that exosomal circHIPK3 is highly expressed in hypoxic exosomes secreted from cardiomyocytes. Silencing of circHIPK3 is associated with increased levels of apoptosis, ROS, MDA, and proapoptotic proteins in cardiac microvascular endothelial cells (CMVECs) (100). The upregulated circHIPK3 sponges miR-29a, an apoptosis-suppressing miRNA, to de-repress the expression of IGF-1, and subsequently regulates the oxidative damage in CMVECs.

## Conclusion

In conclusion, circRNAs are identified as new players to participate in the process of human diseases. Although, the biogenesis and molecular mechanism of circRNAs are still not fully understood, emerging evidences have demonstrated that these circular RNA molecules are broadly present in mammalian cells with different regulatory functions. The heart is composed of a variety of cell types that interact with each other through direct contact or paracrine signaling. More and more studies have proved that circRNAs are involved in the process of cardiac remodeling and have important regulatory functions in cardiomyocytes, endothelial cells, fibroblasts and immune cells during this disease process. The regulation of circRNAs in different cell types of the heart adds a new layer of regulation to the known gene regulation network of cardiovascular disease. Currently, we are still facing challenges in the study of circRNAs. For example, most of circRNAs can not be knocked out for the loss-of-function study since targeting circRNAs using CRISPR–Cas9 or DNA recombination strategy is likely to affect the splicing or expression of linear host genes. Although, the expression of circRNAs could be knocked down by specific siRNAs, the narrow junction of back-splicing limits the design of siRNA for a portion of circRNAs. Therefore, approaches for circRNA study are urged to be improved, which will lead us to better understand the function of circRNAs in human diseases.

Overall, roles of circRNAs in the pathogenesis of cardiovascular disease still remains largely unknown. Unlike linear RNA molecules, the stability of circRNAs grants the advantage of these circular RNA molecules in therapeutic applications, such as disease diagnosis and transgene delivery in gene therapy. Given that extracellular vesicles or exosomes contain circRNAs, capturing tissue-specific and disease-specific vesicles or exosomes for circRNAs profiling could be a good strategy to identify biomarkers for disease diagnosis. Therefore, deeper and systematic studies of circRNAs in the content of different diseases, such as cardiac hypertrophy and heart failure, is the prerequisite of moving the knowledge of circRNAs into the therapeutic applications against the deadly cardiovascular disease.

## Author Contributions

TY, TL, TD, and Z-PH prepared the manuscript. TY, TL, and TD wrote the main parts of the article and produced graphics. YD and YC reviewed and edited the manuscript. Z-PH drafted the final version of the manuscript. All authors read and approved the final manuscript.

## Conflict of Interest

The authors declare that the research was conducted in the absence of any commercial or financial relationships that could be construed as a potential conflict of interest.
